# An Uncommon Epiglottic Cyst Presentation in an Adolescent: A Case Report and Literature Review

**DOI:** 10.7759/cureus.54634

**Published:** 2024-02-21

**Authors:** Hisham Alrashdan, Khaldoun Alshugran, Osama M Alshiyyab, Jawad F Khasawneh, Ethar N Ibrahim

**Affiliations:** 1 Otolaryngology Unit, Royal Medical Services, Irbid, JOR; 2 Anesthesiology Unit, Royal Medical Services, Irbid, JOR

**Keywords:** children and adolescence, airway obstruction, stridor, vallecula, epiglottic cyst

## Abstract

Epiglottic cysts are benign lesions of the larynx that are relatively rare beyond infancy age. A 17-year-old adolescent male patient presented to the outpatient specialized oropharyngeal clinic with inspiratory stridor, chronic sore throat, and progressive dyspnea symptoms over the past eight months. Examination by a headlight and a tongue depressor showed a large cystic lesion arising from the hypopharynx. A neck computed tomography (CT) scan revealed a 4 cm oval cyst attached to the lingual epiglottic surface. The relatively large epiglottic cyst was drained directly in the clinic and was later removed by microlaryngosurgery with traditional microinstrumentation in a follow-up visit. Subsequent recovery was uneventful. Regardless of the rarity of epiglottic cysts in adolescents, doctors should keep in mind this etiology as early diagnosis and management could spare the patient from life-threatening complications or tracheostomy and unneeded medical costs.

## Introduction

Cysts of the epiglottis (i.e. found on either surface of the epiglottis) are usually asymptomatic and are specifically rare beyond infancy age [1]. If large enough, epiglottic cysts can obstruct the airway, and can potentially be life-threatening, especially when an emergency endotracheal intubation is necessary. Due to the soft nature of the mucus retention cysts, dysphagia, and dyspnoea are late to present, which allows such cysts to grow gradually, concealing impact magnitude over time [2]. Usually, such presentations are either found by the otolaryngologist during routine pharynx examination or by the anesthesiologist during intubation. Here, we present an unusual case of a large epiglottic cyst formation in an adolescent so physicians can keep this etiology in mind during routine examinations. The case is presented as per the CARE guidelines for case studies [3].

## Case presentation

A 17-year-old male patient, a non-smoker, presented to the otolaryngology department with a one-week history of stridor, neck flexion, and head rotation to the left. The patient’s minor complaints started eight months ago when he started to feel a foreign body sensation in his pharynx as well as a change in voice tone. Due to the progressive nature of the cyst growth, the patient ignored it and learned to cope with it till the admission day when stridor and dysphagia had started.

**Figure 1:**
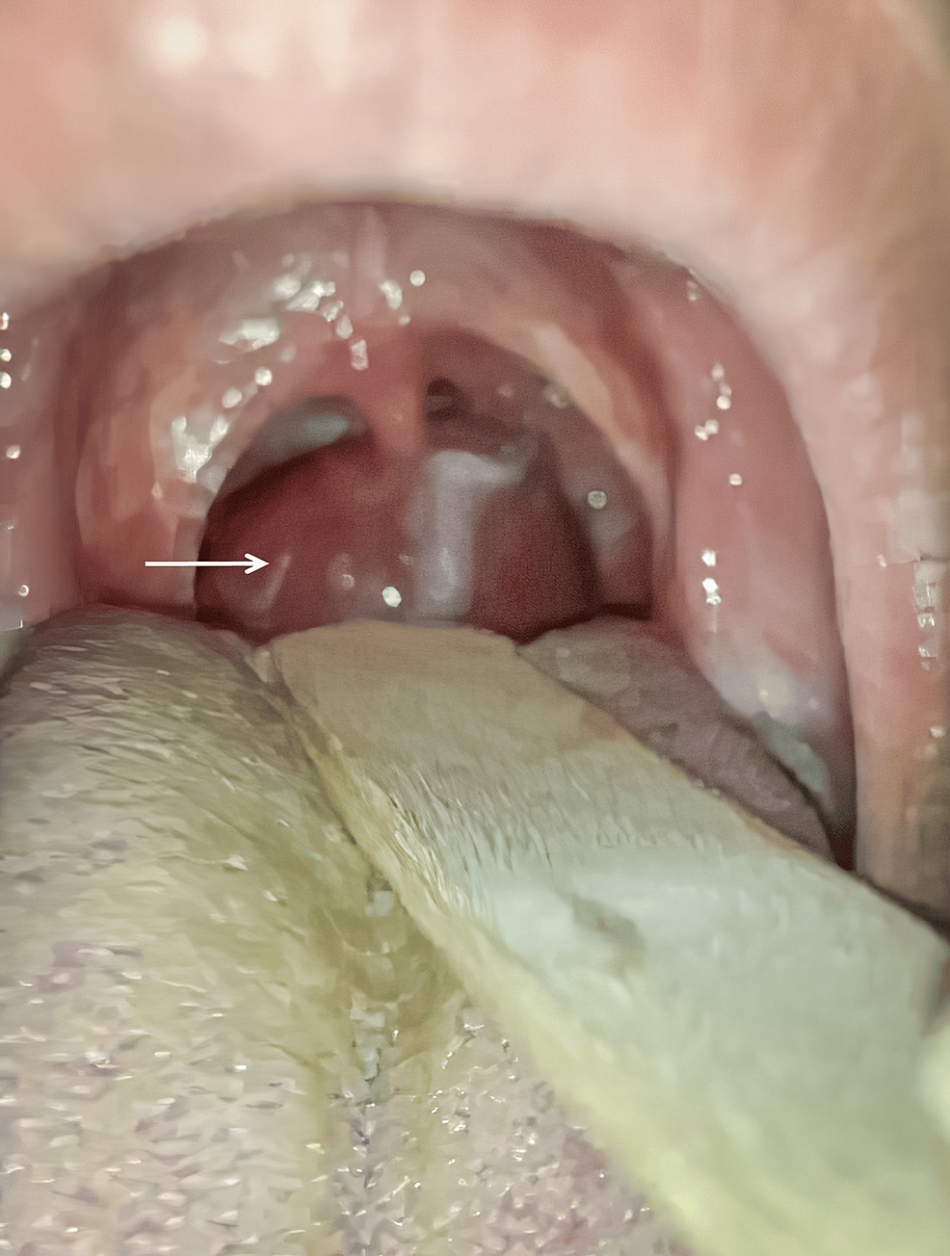
Trans-oral view of the epiglottic cyst.

**Figure 2:**
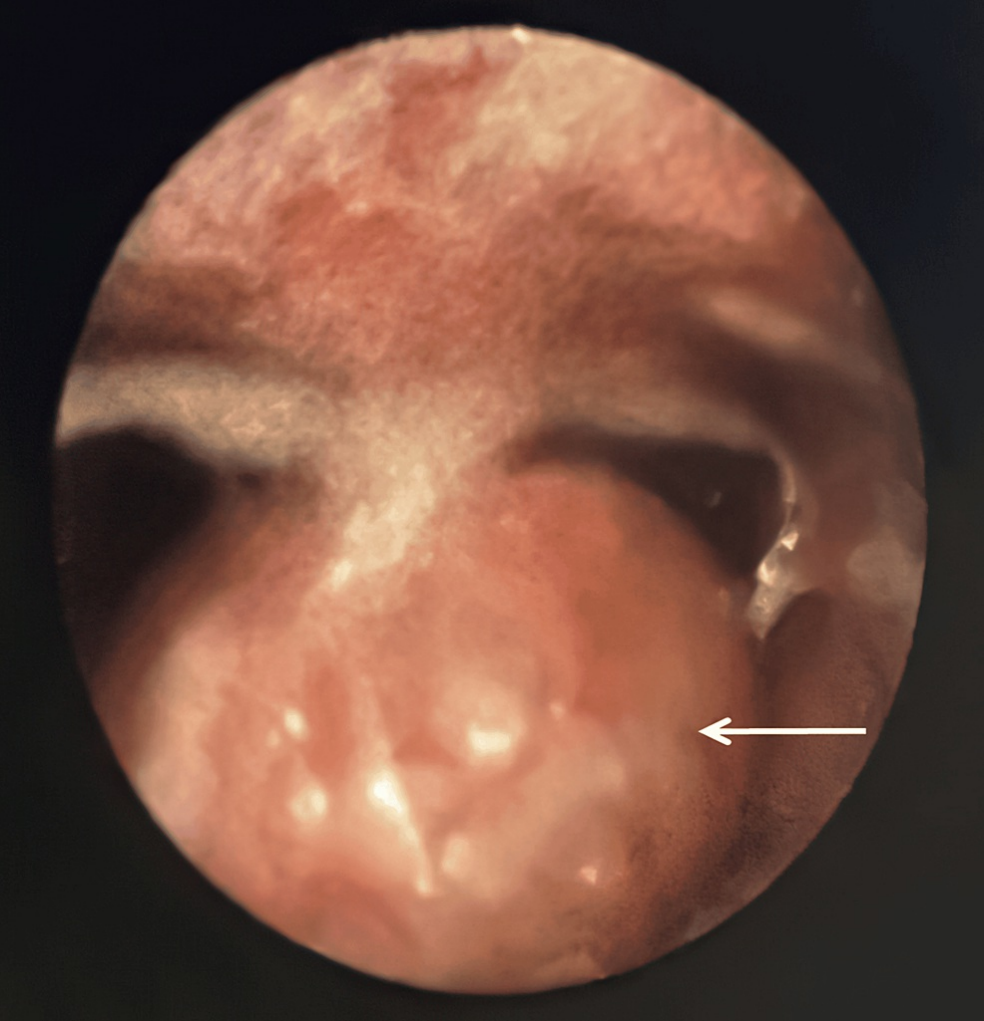
View of the cyst through flexible nasopharyngoscope.

**Figure 3:**
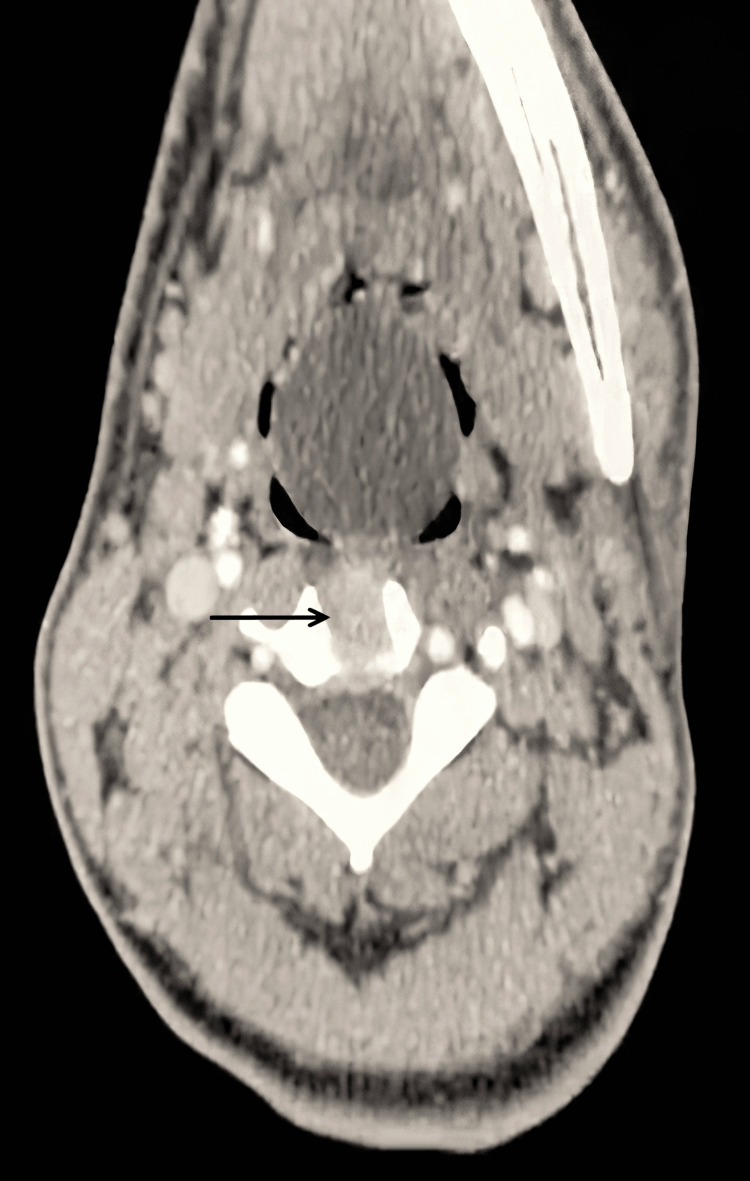
Axial neck CT scan at the hypopharynx level.

Investigation at the clinic with a tongue depressor revealed a ball-like mass with a smooth pinkish surface popping down from his oropharynx (Figure 1). Fiberoptic examination revealed a 4 cm mobile, tense, mass at the level of the epiglottis (Figure 2) and a neck CT scan was done to further investigate the cyst (Figure 3). Investigations confirmed the 4 cm epiglottic cyst originating from the lingual surface of the epiglottis over the left side.

Due to the large size of the cyst, the anesthetist was unsure of the ease of intubation, and the possibility of an emergency tracheostomy was considered, but the decision was made to drain the cyst at the clinic with a 21G needle and do an elective surgery shortly later. This was done following Sugita et al.'s management protocol [4]. Using suction cautery and dissector, about 40 cc of pinkish mucous-like fluid was drained from the cyst. Immediately after the drainage, the patient regained normal breathing and was also able to start a regular diet.

The patient was scheduled for surgery four days later to excise the epiglottic cyst with microlaryngosurgery using traditional microinstrumentation, and direct laryngoscopy and microscope were used for precise excision. The excised cyst was sent for histopathological analysis. The patient was kept overnight for monitoring and was discharged the next day without any medications. Two weeks later, the histopathology report revealed benign squamous epithelium with an underlying cystic lesion. The cyst had not recurred at the time of the six-month follow-up.

## Discussion

Any area of the larynx could give rise to a cyst, but it rarely happens on the lingual surface of the epiglottis or the vocal cords in adolescence; while epiglottic cysts causing airway obstruction have been documented for neonates and infants, their occurrence in adolescents has seldom been reported, suggesting their rarity, despite the absence of precise incidence data [1]. Epiglottic cysts are usually caused by obstruction of the outflow from the larynx mucous glands [5]. Variations of the cyst size and age of presentation lead to different manifestations ranging from sore throat to sleep apnea or stridor. However, an epiglottic cyst with a late diagnosis may complicate into an epiglottic abscess [6]. Different radiological investigations may be done to support the diagnosis of epiglottic cysts; they can be observed as a well-defined soft tissue arising from the epiglottis in a lateral neck X-ray, or as a soft tissue fluid-filled mass, with rim enhancement (if infected) using CT scan and physical examinations. They appear as “hypointense” on T1-weighted MRI images and “hyperintense” on T2 images. Acute epiglottitis is mentioned in the differential diagnosis, but a CT scan would initially reveal epiglottis edema rather than a cyst formation. Another differential diagnosis is laryngeal hemangioma which demonstrates intralesional phlebolith on CT scan with contrast enhancement. Treatment depends upon the size and presenting symptoms. Asymptomatic small cysts can be observed; otherwise, endoscopic deroofing, marsupialization, or excision are all feasible options depending on the specific case on hand [7]. A CO2 laser is a good companion in surgery due to the superior hemostatic effect, and aspiration may ease the procedure [8].

A targeted literature review was conducted to identify case reports similar to ours. The parameters used for this literature search are detailed in Appendix A. The search resulted in a total of 14 articles, of which two were excluded since they were out of scope [9,10]. Twelve articles were finally identified as relevant and listed in Appendix B [4,11-21]. Cases reported were from around the world (e.g. North Korea, United States, China) yet no reports were found for the Middle East. The literature indicated that the management of the situation differed slightly, with some adopting approaches similar to ours, while others utilized a laryngoscopic CO2 laser. In one report, the case was similarly managed using a laryngoscopic CO2 laser [12]. In another report [17], the team adopted a slightly different management approach where transoral fiberoptic intubation was done before the endoscopic excision of this cyst was accomplished. In the United States, a case was handled where transoral fiberoptic intubation was performed prior to the endoscopic removal of the cyst [17]. Further iterative referencing and reference checking revealed another epiglottic cyst case study which was also incidentally diagnosed during a routine video fluoroscopic swallowing study [22]. This confirms the need for physicians not to exclude this diagnosis during regular screening. All studies reported uneventful postoperative course.

## Conclusions

In this report, we presented one of the first cases of epiglottic cyst presentation in an adolescent from the Middle East and North Africa region. Symptomatic epiglottic cysts often cause airway obstruction and may be mistaken with other airway obstructive diseases; hence, regardless of the rarity of epiglottic cysts in adolescents, doctors should keep in mind this etiology as early diagnosis and management could spare the patient unwanted complications or tracheostomy.

## Appendices

 Appendix A

**Table 1:** Targeted literature review parameters.

Parameter	Details
Database	PubMed
Literature review period	1955 to July 2022
Inclusion criteria	Case reports on human subjects with epiglottic cysts
Keywords	("Epiglottic" OR "Epiglottis" OR "Epiglottic Cyst" OR "Epiglottic Cysts" OR "Epiglottic abscess" OR "Epiglottic abscesses") AND ("cyst" OR "cysts") AND ("case study" OR "case report" OR "case series")
Search location	Title and abstract

Appendix B

**Table 2:** Identified epiglottic cyst case reports on PubMed published between 1995-2022.

Title	Journal	Publication Year	First Author
A retrospective case series of anesthetic patients with epiglottic cysts	Anesth Prog	2021	Takaishi K [14]
Epiglottic cyst incidentally discovered during screening endoscopy: a case report and review of literature	Korean J Fam Med	2014	Lee SH [11]
Unusually giant sublingual epidermoid cyst: a case report	Iran J Otorhinolaryngol	2016	Nishar CC [15]
Difficult endotracheal intubation due to an undiagnosed epiglottic cyst: a case report	Korean J Anesthesiol	2009	Lee JH [16]
An awake double lumen endotracheal tube intubation using the Clarus Video System in a patient with an epiglottic cyst: a case report	Korean J Anesthesiol	2014	Seo H [18]
Multiple epidermal inclusion cysts of epiglottis	Kathmandu Univ Med J (KUMJ)	2021	Kc AK [19]
Epiglottic cyst causing dysphagia and impending airway obstruction	Am J Otolaryngol	2015	Collins AM [12]
Spindle cell lipoma of the larynx: a case report	Medicine (Baltimore)	2020	Qin-Ying W [20]
AirWay Scope™ for difficult ventilation in a patient with epiglottic cyst	Anesth Prog	2018	Sugita T [4]
Laryngocele and epiglottic cyst as rare causes of obstructive sleep apnea	Sleep Breath	2009	Araz O [21]
Childhood airway manifestations of lymphangioma: a case report	AANA J	2004	Thompson BM [22]
Acute apnea caused by an epiglottic cyst	Int J Pediatr Otorhinolaryngol	1998	Dahm MC [23]
